# Effects of Disease on the Teeth

**Published:** 1860-09

**Authors:** Abr. Robertson


### EFFECTS OF DISEASE ON THE TEETH.

BY ABR. ROBERTSON, D. D. S., M. D.

(Concluded.)

The general rule, then, is that medicines do not injure the teeth—the rare exceptions proving the rule that they may sometimes do so; and the just inference is, that where this may be the case, care should be used so to administer such medicines as most effectually to prevent it.

The question now recurs, why do teeth decay more frequently and more rapidly during the sickness than at other times?

For the better elucidation of this question, it becomes necessary to inquire what is sickness? I here use this word as a general term for all kinds of diseases. What are its causes? How do the causes act to produce it, and what are some of its effects?

For our present purpose it may be enough to say, that disease or sickness is any disturbance or interruption of all or any of the functions of the living body.

The principal recognized causes of these interruptions and changes of the functions which constitute disease, are indigestible and all the various unwholesome and improper articles of food; malaria, appreciable or unappreciable; sudden changes of temperature; great changes of the state of the atmosphere, electrical and hygrometrical; various emotions of the mind, as grief, fear, anger, and the like; and various viruses and other poisons.

They act upon the system chiefly, if not entirely, in two ways; either by directly mixing with and altering the composition of the blood, as where virus is directly introduced into that fluid; or by some shock or irritation of the nervous system.

The most uniformly observable manner, if not an invariable mode in which all these agencies affect the functions of the living body, is by altering the various secretions. The sweat, the urine, the bile, the gastric and pancreatic fluids, the saliva and the milk, some or all of them have been found more or less altered in most diseases; and the blood, from which all the secretions are derived, is supposed by many always to be changed in disease. It is at least evident that shocks to the nervous system, from purely mental causes, sometimes most wonderfully affect the secretions. Repeated instances have been recorded where a fit of anger has produced such a change in the milk of mothers as to cause the death of their infants in a very short time after partaking of it. I have seen a most obstinate and intractable diarrhœa produced by sudden and excessive grief, causing primarily great acidity of the stomach, and this acid acting most markedly and unmistakably as an irritant on the bowels. But as it is the saliva only—including, of course, the secretions of the salivary glands and buccal mucous membranes—that comes in contact with the teeth, or at most, that remains in contact with them, we shall confine our present inquiries to the changes that have been observed in that secretion; and after remarking that for many years I have observed that confirmed dyspetics almost uniformly have bad teeth, and that their teeth are decayed in so peculiar a manner that I can almost unvaryingly diagnose dyspepsia by an examination of the teeth—(to this I will refer again before closing)—I shall, therefore, quote somewhat freely what has been recorded by others, of the changes produced in that secretion by various diseases.

Muller's Elements of Physiology, page 362, says: "The testimony of many physiologists is, that the saliva is alkaline, but that it may vary from alkaline to acid under many and trifling circumstances."

Lehmann's Chemical Physiology says: "The secretion of the oral mucous membrane has an alkaline reaction. Human mixed saliva, [secretion of the salivary glands and buccal mucous mmbrane] "in an abnormal state has an acid reaction, and commonly in a fasting state. It is most commonly found acid in irritations of the primæ viæ."

Carpenter's Physiology, fifth American edition, page 412, says: "The reaction of the saliva is usually alkaline, that of the principal gland being always so in health; while that of the buccal mucous membrane is acid; so that when the former predominates, as is always the case when food is being masticated and digested, the saliva of the mouth is alkaline, while when the latter is more abundant as is often the case during the interval of digestion, at the slow rate at which the salivary glandulæ then pour forth their product, the buccal saliva is frequently acid."

Simon's Chemistry of Man says, page 295, of human saliva: "When perfectly normal its reaction is alkaline;" also, page 301, *ibid.:* morbid saliva sometimes cantains free acids; this is most commonly lactic acid, but in some cases acetic acid is likewise present. I have frequently found acid in acute rheumatism and in cases of salivation. According to Donne the saliva has an acid reaction in all cases of irritation and inflammation of the stomach, in pleuritis, encephalitis, intermittent fever, acute rheumatism, uterine affections, and amenorrhœa. Brugatelli detected oxalic acid in the saliva of a phthisical patient.

Draper's Physiology says, page 44, that "the saliva is alkaline; but in morbid conditions, an acid reaction is by no means infrequent; it has commonly been observed in intestinal inflammation, acute rheumatism, intermittent fevers."

Thus the testimony of physiologists is unanimous and conclusive that the saliva, under various circumstances, and in many if not most diseases, is changed from its normal alkaline to an acid reaction. Some of them state, as we have seen, that the saliva is usually acid during fasting. This condition usually occurs, to a very great extent, in most cases of sickness of such severity as to require continued active medication.

While, then, the patient is sick enough to require medical treatment, whether from the direct effect of the disease in altering the secretions, or from the fasting consequent upon the sickness producing that effect, or both, his teeth are constantly bathed in almost the only kind of article that has, or can have, any deleterious effect upon them.

Although this acidity may be but slight in degree, it is frequently long-continued and constant, and therefore produces and must produce serious effects; and to my mind, both from theory and long-continued observation, is satisfactory and conclusive cause for the excessive ravages of caries of the teeth during sickness.

But how, I may be asked, does it especially manifest its effects?

Let us take as an example a case of dyspepsia already alluded to, especially as the effects are usually more distinctly marked than in any other kind of disease. For here the acidity of the oral secretions is generally most excessive; and, besides this, such patients are peculiarly liable to great acidity of the gastric secretions, and this they frequently regurgitate into the mouth, and often so strongly acid as to produce at the time a very sensible and disagreeable effect upon the teeth—or what is popularly called "setting the teeth on edge." In such cases, as might be expected, the teeth, if perfectly formed and sound before, will be found most decayed about their necks, that is, just at the margins of the gums; for there in such cases this acid finds most ready lodgment, and is kept most constantly in contact with the tooth; and there, too, the enamel is thinnest, and, therefore, affords the least protection to the body of the tooth; and there, consequently, what enamel there is, will be found to be removed in large patches from many and even all of the teeth, and frequently softened and rendered friable to a much greater extent. If the teeth are already decayed, if even so slightly as to be unobserved by non-professional eyes, or if they have the least imperfections in their formation, as very frequently happens, a want of perfect union of the enamel at the lines of union of that substance from the different points of its deposit, though these unprotected interstices may be ever so small, even so slight as scarcely to show the finest dark-colored lines, they afford similar lodgment for this acid, and also a most favorable opportunity for its fullest and most ready action, and often producing, as every dentist must have seen, whether he recognized the cause or not, most disastrous consequences.

So, too, in all other diseases which change the saliva from an alkaline to an acid reaction, this effect is produced on the teeth in the same manner, and to an extent just in proportion as the saliva is rendered more or less acid, and to the length of time that such change continues.

From these facts, then, we may fairly deduce this rule: Most diseases injuriously affect the teeth; while the exception is that some may not.

What has been said thus far of the agency of diseases in causing decay of the teeth relates to adult life, or at least to their effects upon the teeth after they have erupted through the gums. But there is another time and manner in which diseases most seriously affect the teeth. This is during adolescence—while the teeth are in a state of formation within the gums, where no medicine can act directly upon or come in contact with them. Thus, any disease at this stage of life which interrupts or suspends the functions of nutrition may prevent them from receiving a sufficient supply of the appropriate materials for their full and perfect development. From this cause I have seen a whole set of teeth, otherwise well enough formed, with only the slightest traces of enamel upon them; and it is not very uncommon to see teeth where there is but very little enamel on or near their cutting edges or grinding surfaces, where higher up on the crowns of the same teeth it is of normal quantity and quality, showing that the process of nutrition or of supply had from some cause, for a time, been suspended, and afterwards restored. This effect is also made manifest by the frequent observation that some of the teeth of the same individual may be found decayed or imperfect in their construction, while the others are sound and perfect—the decayed or imperfect ones and the sound being in pairs, and of the same class, showing that the same cause that acted deleteriously on one, had the same action on the others that were in the same stage of their formative process. So true is this that I have often been able to diagnose at what age of the individual a previous "fit of sickness" had taken place, without any previous knowledge of the facts, and by simply observing in what part of the mouth such decayed teeth were situated.

Having now shown that medicines, judiciously exhibited and properly administered, do not, and generally cannot, injure the teeth, and that many if not most diseases do, and of necessity must injure them more or less, I cannot refrain from adding that the injudicious exhibition and improper administration of medicines, and more especially the drenching themselves with quack nostrums, to which our people are so strangely prone, as uniformly do and must produce ill effects on these organs. And so, too, and in the same manner, may the eating of candies, or of sugar, or of the very staff of life, bread, or the drinking of pure cold water; *i. e.*, by the taking of them at such improper times and in such improper quantities, and at such irregular intervals as to disturb the functions of the stomach and nervous system; thus injuring the health and changing the character of the secretions. Hence, a safe general rule in relation to quack nostrums is, that any time is an improper time, and any quantity is an improper quantity. And this is true at all stages of existence, from earliest youth to decrepit age. But medicines, when so administered as to secure the object for which they are given—the restoration of the secretions from an abnormal to their normal condition, the restoration of the body from disease to health—instead of injuring the teeth, protect them from injury.

